# Behcet's disease: Diagnosed as isolated recurrent oral aphthae; a case report

**DOI:** 10.1016/j.amsu.2022.104327

**Published:** 2022-08-24

**Authors:** Maysoun Kudsi, Naram Khalayli, Amr Allahham

**Affiliations:** a Faculty of Medicine, Damascus University, Damascus, Syria; b Faculty of Medicine, Syrian Private University, Damascus, Syria

**Keywords:** Behcet's disease, Oral aphthae, Dapsone treatment

## Abstract

**Background:**

Behçet's disease is a multisystem autoimmune disorder of unknown origin, characterized by frequent oral and genital ulcerations, ocular and cutaneous lesions, arthritis, and it may also involve the gastrointestinal, and central nervous system. In this paper we discuss a case of Behcet syndrome presenting with isolated oral aphthous initially and spotlight on the clinical features and treatment.

**Case report:**

A case of a 32-year-old male with repetitive oral aphthous on the tongue and the inner cheeks for four days with a history of frequent recurrence. The patient responded to topical triamcinolone acetonide and 5–10 mg/day prednisone. Three weeks later, there was a recurrence of oral aphthous, genitalia lesions, and blurred vision with left eye redness. A uveitis diagnosis was made, and the patient was placed on 75mg (1mg/kg) prednisone with a resultant response. There was another recurrence with all the previous symptoms, so the decision with Dopson treatment was made. The patient was placed on Dapsone, resulting in complete resolution of the lesions with no reproduction within and after six months of several recall visits. The diagnosis of Bechet syndrome was made.

**Discussion:**

Aphthous ulcers may be seen in a variety of disorders, while recurrent and persistent >6 weeks, individual non-healing ulcers require a review of the diagnosis. Behcet disease usually appears 2–3 years after oral and genital ulcers, but oral aphthae may be its first manifestation in 10–20% of cases. AS oral ulcers are among the common complaints of the polyclinics-patients, it is recommended to educate the health professionals to diagnose these unusual cases in the first instance, thus avoiding underdiagnosed and the time management to reduce complications and improve the quality of life in these patients.

**Conclusion:**

A case of Behçet's disease in a 32-year-old male who presented initially with oral mucosa and later caused uveitis and genitalia aphthae.

## Highlights

•Background: A case of Behcet syndrome presenting initially with isolated oral aphthous.
• Case Report: A 32-year-old male with recurrent oral aphthous. A complete resolution of the lesions with no reproduction.
• Conclusion: A case of Behcet's syndrome affected the oral mucosa and later caused uveitis and the genitalia aphthae.
• Keywords: Oral aphthae, Behcet's Disease, Dapsone treatment .

## 1 Introduction

Behcet disease, also known as Silk Road disease, is a chronic multisystem autoimmune syndrome of unknown origin [1]. Its prevalence ranges between 0.1/1000 and 1/10,000, with a higher incidence in Asia, Mediterranean countries, and Japan; it remains rare in other countries such as the Americas [2]. Its peak incidence in the third decade, with women predominance [3]. It represents an autoimmune reaction triggered by an infectious agent in a genetically predisposed person [4]. Genetic studies should that HLA-B*51 is a significant genetic risk factor [5]. It affects all organs such as the skin, the eye, the blood vessels, gastrointestinal tract, lungs, central nervous system, heart, and others [6]. Painful recurrent mouth ulcers may occur at some time in all patients. They begin as raised, round lesions that quickly turn into painful ulcers. They're often the first noticed symptom and may occur 2–3years before any other symptoms of Behcet disease appear. In addition, they may be the first manifestation of the disease in 10–20% of cases [7,8]. Multiple criteria have been suggested as guides for diagnosis, although some cases remain undiagnosed [9]. The American Criteria of Rheumatology (ACR) put five criteria for diagnosis: oral aphthae, genital aphthae, skin features, eye involvement, and positivity of pathergy test [10].

We present a case of Behcet disease in a male patient who presented initially with the frequent, persistent isolated occurrence of oral aphthae.

This case series has been reported in line with the SCARE Criteria [23].

Research Registry unique identifying number (UIN) 7991 [24].

## 2 Case report

A 32-year-old married male hand worker presented to the Rheumatology clinic as consultation from the Dental clinic, Damascus University, with a two-year history of frequent recurring oral aphthae. No considerable family history nor psychosocial history has been reported by the patient. He was on topical steroid-triamcinolone acetonide 0.1%, colchicine 1mg daily for four weeks. Following several follow-up visits, the ulcers healed. After six months, the patient presented with recurrent aphthous ulcers on the tongue and inner cheeks for four days.

**Fig. 1:**
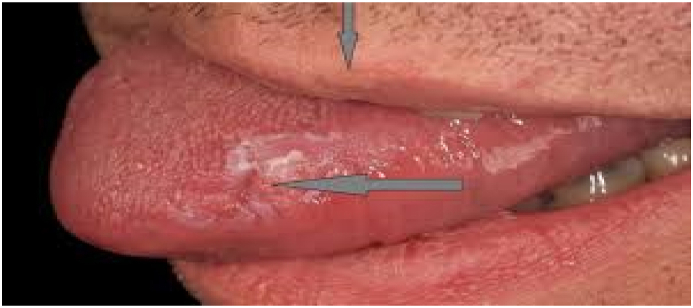
Major Aphthous ulcer on the tongue.

**Figure-2:**
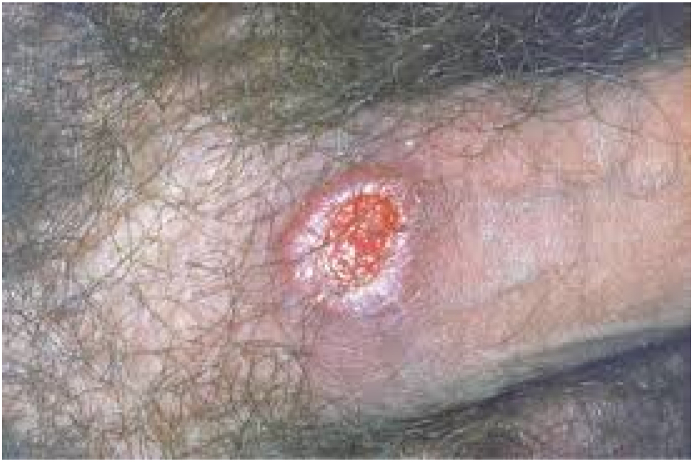
genitalial aphthae.

On examination, a pale oval floor with erythematous margins was found. He was placed on topical triamcinolone acetonide twice daily for seven days, Prednisolone 10mg daily for seven days followed by 5 mg for the next five days, and tetracycline mouth washes four times daily for seven days. The patient responded well with the healing of the ulcers after the steroid therapy. However, just after two weeks, there was a recurrence of the oral aphthous ulcers with erosions, genitalial lesions, and blurred vision with the red left eye (Fig. 1, Figure-2).

According to the ophthalmology consultation, acute anterior uveitis was seen. Our patient was placed on 75mg/OP/day (1mg/kg) Prednisone for seven days, tapering down gradually by 5 mg after every seven days; continue the topical triamcinolone acetonide. There was magnificent healing of the ulcers and erosions, uveitis, and genital ulcers after two weeks of treatment. Again, after four weeks, there was a relapse with manifestations of sore throat, genitalia ulcerations, redness, left eye pain and poor vision, and associated abdominal pain. Based on these findings, the diagnosis of Behcet's syndrome was made according to the ACR criteria. To avoid the side effects of using systemic steroids, the patient was placed on Dapsone 25 mg twice daily for one week and continued with the topical triamcinolone acetonide. The patient was also referred to the ophthalmology clinic, where he was managed for uveitis. Complete resolution of all clinical manifestations happened, with no recurrence within six months of several visits.

## 3 Discussion

Bechet's disease is a multiorgan disease that affects men more than women [11], with a peak incidence in the third decade [12]. Our patient is a male presented in his fourth decade. As there were no previous studies about age and gender in Syria, more extensive studies would help determine the concordance with the international studies.

The American Criteria of Rheumatology (ACR) put five criteria for diagnosis of Bechet disease, which include the presence of recurrent oral ulceration along with any two of the following: recurrent genital ulceration, ocular lesions, and cutaneous lesions, or positive pathergy test results [10]. Almost everyone with Behçet's disease develops mouth ulcers. Thus, these ulcers look the same as typical mouth ulcers, but can be more painful, more frequent, multiple, and larger, found on the lips, tongue, and inside of the cheek, and usually appears 2–3 years before the onset of Bechet disease, as its first manifestation in 10–20% of cases [6,7].

Our patient had an oral aphtha which was the first presentation, then he had genital ulcer and uveitis, which made the diagnosis of Bechet's disease.

Many treatments have been used to decrease morbidity and mortality [13]. The treatment includes steroids, immunosuppressive, and biological therapies [14].

Corticosteroids can help reduce the inflammation associated with Behçet's disease. Depending on the specific symptoms being treated, corticosteroids are used as topical, oral, and injections [1,6]. Topical corticosteroid side effects are uncommon, but long-term use may lead to thinning of the skin. The long-term use of oral corticosteroids is associated with more side effects; some of them are serious [15].

To avoid our patient steroids side effects, we used Dapsone. Dapsone has been reported to treat mucocutaneous manifestations [6], reducing the frequency and duration of oral and genital ulcers [16]. Although it has side effects [17], our patient had no adverse effects.

Some diseases such as erythema multiforme, Steven-Johnson syndrome, recurrent aphthous stomatitis, herpetic stomatitis, and complex aphthosis may mimic Bechet disease [6].

Erythema multiform shows hemorrhagic and crusting ulceration with multiple erythematous patches on the skin and mucosa [18]. Steven Johnson's syndrome is characterized by polymorphic skin lesions, acute blisters, and mucous membrane erosions [19]. Complex aphthosis is an uncommon‚ persistent type of recurrent aphthous stomatitis which may also be associated with systemic diseases [20].

In medical literature, limited case reports have been published about oral ulcers as a first manifestation of Bechet disease, which was first described in 1981 (21). In our case, the oral ulcers were presented 2–3 years before the diagnosis of Bechet disease. This extended time of misdiagnosis may lead to inappropriate treatment, and thus undesired complications and decreasing the quality of life in these patients. Within two weeks, he had a genital ulcer, uveitis, which may cause blindness, and gastrointestinal involvement, which is considered an essential manifestation of active disease; meanwhile, the previous case reports, the duration was about one year till the other symptoms like genital ulcers, conjunctiva inflammation, erythema nodosum, and skin ulcers appeared [6,21,22]. No data are available about the economic impact of this condition, but the high rate of morbidity and mortality of the disease, due to the major organ involvement, should be considered.

## 4 Conclusion

We report a case of Behcet's syndrome presented initially with just recurrent oral aphthae. Awareness of this rare condition should be thought of by Healthcare professionals.

## Ethical approval

This study was ethical approved by faculty of medicine, Damascus University.

## Sources of funding

The study has no funding of any sources.

## Author contribution

All the authors of this paper have contributed to the study concept, data collection, data analysis, and writing the paper.

## Conflicts of interest

No conflicts of interest.

## Registration of research studies

1. Name of the registry: Behcet's Disease: Diagnosed as Isolated recurrent oral aphthae; A case report
2. Unique Identifying number or registration ID: researchregistry7991
3. Hyperlink to your specific registration (must be publicly accessible and will be checked): Browse the Registry - Research Registry

## Guarantor

Amr Allahham, amroapple98@gmail.com.

Maysoun Kudsi, MayKuds@gmail.com.

## Consent

Written informed consent was obtained from the patient for publication of this case report and accompanying images. A copy of the written consent is available for review by the Editor-in-Chief of this journal on request.

## Annals of medicine and surgery

The following information is required for submission. Please note that failure to respond to these questions/statements will mean your submission will be returned. If you have nothing to declare in any of these categories then this should be stated.
